# The Promotion of Creativity of Vocational College Students: The Role of Parent-Child Relationship, Emotional Intelligence, and Grit

**DOI:** 10.3389/fpsyg.2021.765444

**Published:** 2021-11-16

**Authors:** Yushen Wu, Yubin Wu, Daohan Chong, Wen Zhang

**Affiliations:** ^1^Faculty of Psychology, Beijing Normal University, Beijing, China; ^2^Hakka Studies College of Gannan Normal University, School of History Culture and Tourism, Gannan Normal University, Ganzhou, China; ^3^Shandong Water Conservancy Vocational College, Rizhao, China

**Keywords:** parent-child relationship, creativity, emotional intelligence, grit, vocational college student

## Abstract

**Objective:** To examine whether emotional intelligence played a mediation role in the association between parent-child relationship and vocational college student’s creativity, and whether grit moderated this mediating process.

**Methods:** 663 vocational college students participated in this study and completed four questionnaires at three time points, which included measures of parent-child relationship, creativity, emotional intelligence, and grit.

**Results:** (1) Emotional intelligence mediated the relationship between parent-child relationship and vocational college student’s creativity; (2) grit moderated the mediating role of emotional intelligence between parent-child relationship and vocational college student’s creativity.

**Conclusion:** Parent-child relationship had both direct effects on vocational college student’s creativity and indirect effects through emotional intelligence. Grit moderates the effect of emotional intelligence on vocational college student’s creativity.

## Introduction

In recent years, technological innovation is an important engine for national development. To cultivate innovation, it is important to improve individual creativity, because creativity can help promote the adjustment of the country’s economic structure and continuously enhance the new impetus for economic development. Expansion of higher education has become commonplace in developed economies as a well-educated and highly skilled population, as human intellectual capital, is considered a core aspiration to securing economic advantage in the global knowledge market (Parry, 2009; Gale and Tranter, 2011; Griffioen and de Jong, 2013; Avis and Orr, 2016). Creativity is an important indicator for the cultivation of innovative talents in colleges and universities, especially for vocational college students. The future career of vocational college students is mainly based on technology, and the cultivation of their innovation is an important part of the country’s innovative talent reserve (Xue and Li, 2021). Therefore, in the era of advocating innovation, it is necessary to explore the mechanisms that relate to individual creativity in order to effectively develop individual creativity and enhance the creative ability of vocational college students. Based on the person-environment fit theories of creativity, this research explores the impact of parent-child relationship as environmental factors, emotional intelligence, and grit as individual factors on creativity of vocational college students.

Creativity is not determined solely by personality traits, thinking habits, or abilities, and the environment in which the individual is located, and it is determined by the interaction of various factors, such as researchers believe that creativity comes from the interaction of individuals, fields, and domains (Csikszentmihalyi, 1999). Runco (2007) emphasizes the important impact of the interaction between human and environment on innovation performance. Therefore, it is particularly important to understand creativity from the person-environment fit theories, the theory believes that the interaction between the individual and the environment is the essential element that affects individual creativity (Sen et al., 2014). So this study evaluates creativity from both personal and environmental aspects. Parent-child relationship is an important environmental factor affecting individual development, research has shown that an important factor affecting the development of individual creativity is the parent-child relationship (Li and Shi, 2004). A good and harmonious parent-child relationship plays an important role in the individual’s psychological and behavioral development (An and Cooney, 2006; Brook et al., 2006). Being in a close and harmonious parent-child relationship for a long time will provide more possibilities for the development of individual creativity, studies have shown that early family environment, such as parent-child relationship, is related to higher level of individual creativity (Zhang D. et al., 2018). Attachment theory points out that a good parent-child relationship is a protective factor for the development of individual behavior and various abilities (creativity) (Bowlby, 2010; Padilla-Walker et al., 2016). Although the relationship between parent-child relationship and creativity is relatively clear, the internal mechanism of action remains unclear.

Ecosystem theory points out that individual characteristics and environmental factors have important impacts on individual development (Bronfenbrenner, 1989). Emotional intelligence refers to an individual’s ability to perceive, express, and manage his or her own emotions, and to recognize others’ emotions, and to use the information to guide his or her thinking and actions (Joseph and Newman, 2010). It is also an individual’s ability to manage emotions and communicate with others in life (Joseph and Newman, 2010). Attachment internal working model indicates that parent-child relationship can affect the individual’s perception, that perception by providing their own emotions, understand the emotions of others, manage their emotions, assessing emotions of others pattern and thus enhance the emotion regulation and social adaptability of individuals (Bowlby, 2010). The empirical results also confirmed that good parent-child relationship will significantly enhance emotional intelligence (Bruce, 2014; Wang et al., 2019). On the other hand, individual’s emotional intelligence will also affect the development of creativity, the higher the level of emotional intelligence, the stronger their creativity (Rodrigues et al., 2019). Emotional information processing theory also pointed out that, for each processing needs, individuals with high emotional intelligence are accustomed to using effective emotional management strategy while maintaining individual positive emotions, thus enhancing their creativity fundamentally (Côté, 2014). Brackett et al. (2011) also pointed out the individual with high emotional intelligence will respond effectively to the everyday environment and to take creatively. It can be inferred from this that the parent-child relationship may affect the development of creativity by affecting the development of individual emotional intelligence. Therefore, this study proposes the hypothesis that the emotional intelligence of vocational college students plays a mediating role in the influence of parent-child relationship on individual creativity.

Mediating variables have a clear explanation of “commonness and process,” but the question “personality or conditions” between independent variables and the outcome variable lacks of attention. In this study, emotional intelligence can explain the “process” between parent-child relationship and creativity, but it cannot explain whether there are other variables moderating this “process.”

The individual environment interaction model points out that individuals who grow up in different environments may show different behaviors or problems even if they have the same characteristics and abilities (Neufeld et al., 2006). Grit is a positive psychological quality, which shows perseverance of effort and consistency of interest. Study found that the quality of grit is important for the healthy growth of the individual (Duckworth et al., 2007). Resilience protection model theory points out that protective factors can effectively alleviate the negative impact of risk factors on their development, and promote the healthy development of individual (Garmezy et al., 1984). Grit, as a protective psychological quality, may also play the same role, and individuals with high level of grit may try their best to overcome difficulties in the process of achieving long-term goals, especially in challenging and creative work or tasks. The empirical study also found that grit can effectively alleviate the negative effects of negative events on individual adaptation and learning, and enhance positive energy (O’Neal et al., 2016; Lei et al., 2019). In addition, because emotional intelligence and grit are closely related to parent-child relationship, and largely depend on the quality of parent-child relationship to form and develop. Therefore, this study proposes the hypothesis that grit only plays a moderating role in the path of emotional intelligence and creativity.

**FIGURE 1:**
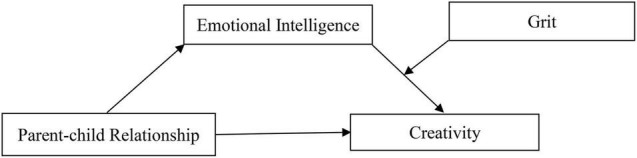
The mechanism effect of vocational college students’ parent-child relationship on creativity.

In conclusion, based on the person-environment fit theories of creativity, this study constructs a moderated mediating model. The main purpose of this study is to explore the mechanism of parent-child relationship affecting individual creativity, to examine whether emotional intelligence mediates this relationship, and whether grit moderates the influence of emotional intelligence on individual creativity. Based on the above theory and hypothesis of this study, a theoretical model of the mechanism effect of vocational college students’ parent-child relationship on creativity was constructed in Figure 1.

## Materials and Methods

### Participants and Procedure

This study randomly selected three vocational colleges in Shandong Province, China, and the survey process was approved by the principal of three vocational colleges. 680 students were randomly selected from these three colleges according to their student id numbers. In order to avoid common method bias, sample data were collected at three time points, and the time interval was 2 months. Parent-child relationship was measured at time point 1, emotional intelligence and grit were measured about 2 months later (time point 2), and creativity was measured about 2 months later (time point 3). We conducted a questionnaire survey with the teacher’s consent before the students started the class, it was done as an assignment for the students, so we recovered 680 questionnaires, the recovery rate is 100%. And we conducted on-site guidance to students throughout the investigation process. However, 17 questionnaires were incompletely filled, and some items were not filled in by the subjects, which were excluded by us, so we obtained 663 valid questionnaires with a questionnaire efficiency of 97.5%. Among them, there are 134 men and 529 women, with an average age of 19.71 years and a standard deviation of 0.97. Sensitivity analysis using G^∗^Power (Faul et al., 2009) revealed that, a study with this sample size can detect medium sized effect in an independent samples *t*-test (α = 0.05, two-tailed): effect sizes = 0.55, with sufficient power (1-β > 0.95).

### Measures

#### Parent-Child Relationship

The family adaptation and parent-child affinity evaluation scale revised (Olson et al., 1979) by Zhang et al. (2006) was used to measure the parent-child relationship of vocational college students. The scale has an affinity with the father-child affinity and mother affinity two dimensions, comprising 20 items, Likert 5-point score (1 = almost never, almost always = 5), The higher the score of the scale, the better the quality of the parent-child relationship of the research subject. This scale was reliable in this study (Cronbach’s α = 0.897), and the confirmatory factor analysis result was χ*^2^/df* = 3.274, *CFI* = 0.969, *CFI* = 0.940, *RMSEA* = 0.059, *p* < 0.001, which showed that the scale had a good structural validity.

#### Creativity

The person–environment fit scale of creativity (PEFSC) compiled by Sen et al. (2014) was used to measure the creativity of vocational college students. The scale contains two factors: individual and environment. Each factor contains 7 items, and each item uses Likert’s 5-point scoring (1 = none, 5 = always). The higher the scale score, the higher the individual’s creativity level. This scale was reliable in this study (Cronbach’s α = 0.96), and the confirmatory factor analysis result was χ*^2^/df* = 3.419, *CFI* = 0.980, *GFI* = 0.955, *RMSEA* = 0.060, *p* < 0.001, which showed that the scale had a good structural validity.

#### Emotional Intelligence

The Emotional Intelligence Scale compiled by Hong Kong scholar Wong and Law (2002) was used to measure the emotional intelligence of vocational college students. The scale contains 16 items, divided into four dimensions: self-emotion assessment, other people’s emotion assessment, emotion management and emotion utilization. Each project uses Likert’s 7-point scoring (1 = completely disagree, 7 = completely agree). The higher the scale score, the higher the individual’s emotional intelligence level. This scale was reliable in this study (Cronbach’s α = 0.95), and the confirmatory factor analysis result was χ*^2^/df* = 2.589, *CFI* = 0.985, *GFI* = 0.958, *RMSEA* = 0.049, *p* < 0.001, which showed that the scale had a good structural validity.

#### Grit

The Grit Scale compiled by Duckworth et al. (2007) was used to measure the grit of vocational college students. The questionnaire contains two dimensions: consistency of interest and persistence of effort, a total of 12 items, using Likert 5-point scoring (1 = very unlike me, 5 = completely like me). The higher the scale score, the higher the individual’s grit level. This scale was reliable in this study (Cronbach’s α = 0.87), and the confirmatory factor analysis result was χ*^2^/df* = 3.210, *CFI* = 0.981, *GFI* = 0.971, *RMSEA* = 0.058, *p* < 0.001, which showed that the scale had a good structural validity.

### Data Analysis

The SPSS 22.0 statistical software was used for descriptive statistics, correlation analysis, and moderated mediating model analysis, and adopted the Bootstrap program for inspection. And AMOS 22.0 was used for confirmatory factor analysis.

## Results

### Common Method Deviation Analysis

The study adopted Haman’s single-factor test to carry out the common method deviation analysis on all the valid data (Podsakoff et al., 2003). As a result, the present study found that there were 11 factors featuring root values greater than one and that the variance of the first one was 32.35%, smaller than the critical value of 40%, which meant that the common deviation method of the study was not remarkable.

### Preliminary Analysis

The results of descriptive statistics and correlation analysis are shown in Table 1. Results revealed that vocational college students’ parent-child relationship had a positive correlation with creativity (*r* = 0.36, *p* < 0.01), emotional intelligence (*r* = 0.37, *p* < 0.01), and grit (*r* = 0.30, *p* < 0.01). Emotional intelligence had a positive correlation with creativity (*r* = 0.57, *p* < 0.01) and grit (*r* = 0.38, *p* < 0.01). Grit had a positive correlation with creativity (*r* = 0.33, *p* < 0.01). It was show that it was necessary to further reveal the internal relationship between the elements.

**TABLE 1:** Correlation coefficients, means, and standard deviations of variables (*n* = 663).

	***M* ± *SD***	**1**	**2**	**3**	**4**	**5**
(1) Gender	–	−				
(2) Age	19.71 ± 0.97	0.22	−			
(3) Parent-child relationship	3.72 ± 0.61	–0.08	–0.07	–		
(4) Emotional intelligence	5.10 ± 0.96	0.11**	0.08	0.37**	–	
(5) Grit	3.14 ± 0.38	0.07	–0.03	0.30**	0.38**	–
(6) Creativity	3.54 ± 0.72	0.13**	−0.11**	0.36**	0.57**	0.33**

**p < 0.01.

### Moderated Mediation Model Testing

Although the moderated effect of grit on emotional intelligence and individual creativity was theoretically described, in order to verify the overall effect of grit, the moderated effect of grit on three pathways of the mediating model was verified separately. According to Wen and Baojuan (2014), three regression equations were tested in this study. Equation 1 tested the moderated effect of grit on parent-child relationship and individual creativity. Equation 2 tested the moderated effect of grit on parent-child relationship and emotional intelligence. Equation 3 tested the moderated effect of grit on emotional intelligence and individual creativity. All predictive variables were normalized in each equation (Garmezy et al., 1984). In addition, previous studies have shown that gender, age are important factors that influence volunteering behavior (Warren et al., 2018; Zhang W. J. et al., 2018), therefore, the present study viewed gender and age were used as control variables.

During the analysis, we found that the variance inflation factor (VIF) of all the predictive variables was less than 10. Therefore, there was no serious multicollinearity problem in this study. As shown in Table 2, parent-child relationship in Equation 1 was positively correlated with individual creativity, and the interaction term of parent-child relationship and grit was not significantly correlated with individual creativity (grit × parent-child relationship). In Equation 2, parent-child relationship was positively correlated with emotional intelligence, and the interaction term of parent-child relationship and grit was not significantly correlated with emotional intelligence (grit × parent-child relationship). In Equation 3, emotional intelligence was positively correlated with individual creativity, and the interaction term of emotional intelligence and grit was significantly correlated with individual creativity (grit × emotional intelligence). In conclusion, we could conclude that emotional intelligence played a mediating role between parent-child relationship and individual creativity, and the mediating effect accounted for 30.54% of the total effect. In order to further tested whether there was a mediating effect, carried out the bootstrap inspection of the 95% confidence interval, if the 95% confidence interval does not contain 0, the mediating effect was significant (Preacher and Hayes, 2004; Preacher et al., 2007). We carried out the bootstrap inspection of the 95% confidence interval, setting the self-sampling number to 5,000, and the 95% confidence interval was [0.165, 0.286], which did not contain 0, therefore, it was indicated that the mediating effect of emotional intelligence exists. In addition, the study also found that grit only moderated the relationship between emotional intelligence and individual creativity, that is, it moderated the latter half of the mediating model.

**TABLE 2:** Moderated mediation model analysis (*n* = 663).

	**Equation 1(outcome variable: creativity)**			**Equation 2(outcome variable: emotional intelligence)**			**Equation 3(outcome variable: creativity)**		
	β	***t***	***95%CI***	β	***t***	***95%CI***	β	***t***	***95%CI***
Gender	0.24	3.87***	[0.12, 0.37]	0.26	3.14**	[0.10, 0.42]	0.15	2.58*	[0.04, 0.26]
Age	–0.03	−0.34*	[-0.06, -0.01]	0.02	1.03	[-0.02, 0.06]	–0.04	−3.23**	[-0.07, -0.02]
Parent-child relationship	0.36	8.24***	[0.27, 0.45]	0.46	8.14***	[0.35, 0.58]	0.19	4.66***	[0.11, 0.27]
Emotional intelligence							0.36	13.59***	[0.31, 0.42]
Grit	0.42	5.98***	[0.28, 0.56]	0.72	7.85***	[0.54, 0.90]	0.14	2.16*	[0.01, 0.27]
Grit × parent-child relationship	0.03	1.45	[-0.01, 0.08]	0.01	0.16	[-0.05, 0.06]			
Grit × emotional intelligence							0.05	2.59*	[0.01, 0.08]
*R* ^2^	0.21			0.23			0.38		
*F*	33.20***			39.30***			67.85***		

*p < 0.05, **p < 0.01, and ***p < 0.001.

**FIGURE 2:**
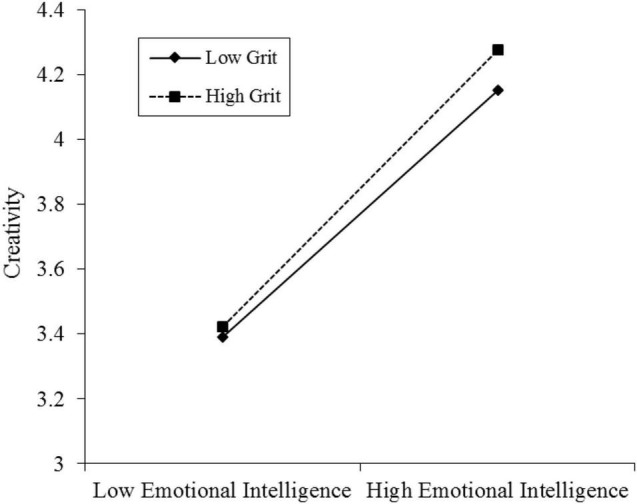
The moderated effect of grit on emotional intelligence and individual creativity.

In order to further explore how grit regulates the relationship between emotional intelligence and individual creativity, a simple slope analysis was conducted. According to the standard of one standard deviation above (below) the mean, the groups with high and low grit levels were divided into two groups. The results showed that with the increase of emotional intelligence, the creativity of both high grit (*B_simple slope_* = −0.24, *t* = −5.73, *p* < 0.001) and low grit (*B*_*simple slope*_ = −0.45, *t* = −10.34, *p* < 0.001) groups increased significantly, but the creativity of high grit group increased more obviously (see Figure 2).

## Discussion

This study builds a moderated mediation model to test the relations of grit and emotional intelligence and parent-child relationship and individual creativity. The study found that emotional intelligence acted as a mediation between the parent-child relationship and individual creativity, in addition, the second half path of the mediation was moderated by grit after controlling gender and age.

### The Mediating Effects of Emotional Intelligence

This study not only verifies that the parent-child relationship has a direct impact on individual creativity, but also finds that emotional intelligence plays in part a mediating role in individual creativity, which indicates that the parent-child relationship not only has a direct bearing on individual creativity, but also has an indirect effect on individual creativity by cultivating individuals’ emotional intelligence.

This study can explain the mediating effects of emotional intelligence from the following aspects. First, emotional intelligence in theoretical terms is the capability of individuals to recognize their own emotions and those of others, discern between different feelings and label them appropriately, use emotional information to guide thinking and behavior, and adjust emotions to adapt to environments (Joseph and Newman, 2010). The broaden-and-build theory of positive emotions suggests that positive emotions broaden one’s awareness and encourage novel, varied, and exploratory thoughts and actions (Fredrickson, 2001). In other words, positive emotions can effectively help individuals develop divergent thinking and develop more novel designs (plans) (Duan et al., 2013). Affective events theory (AET) also suggests that positive emotions can effectively improve individuals’ divergent thinking and further develop more novel designs (plans) in their work or study (Duan et al., 2013). And good emotional intelligence ability can make the individual perceive more positive emotions and have a beneficial impact on the individual’s creativity. Attachment theory suggests that the parent-child relationship can affect the individual’s ability to perceive emotions (Joseph and Newman, 2010). So emotional intelligence is closely related to the family relationship (the parent-child relationship). Therefore, the parent-child relationship can improve individuals’ level of emotional intelligence and further improve their level of creativity. Theories related to attachment and emotion provide the theoretical basis for emotional intelligence acting as an intermediary between the parent-child relationship and individual creativity. Second, emotional intelligence in empirical terms can reinforce positive behaviors such as individual performance (O’Boyle et al., 2011), innovative behavior (Wang et al., 2016), and job involvement (Yan et al., 2018). Moreover, the parent-child relationship can improve the level of emotional intelligence (Belean and Năstasă, 2017; Wang et al., 2019). Hence, this study in both theoretical and empirical terms verifies that emotional intelligence plays a mediating role in the relationship between the parent-child relationship and individual creativity.

### The Moderating Effects of Grit

This study found that grit played a moderating role in the relationship between the parent-child relationship and individual creativity. To be specific: the moderating effects occur at the second half of the mediating chain. In other words, grit has regulated the relationship between emotional intelligence and individual creativity. Results show that grit can significantly improve emotional intelligence’s impact on individual creativity, however, low its level is. According to “protective factor-protective factor” (Cohen et al., 2003), two kinds of protective factors have different effects on individual creativity: the facilitating effect and the excluding effect. However, this study found that grit had an accumulative effect rather than a reductive effect on emotional intelligence, which indicates that grit can help individuals find new solutions and further improve their creativity through interest and unremitting efforts.

### Research Limitations and Future Research

There are some valuable conclusions in this study, but it has its limitations. Firstly, in our sample, over-represent women and under-represent older students, and besides gender and age, we do not collect other demographic characteristics. The study in the future can increase the sample size of man and collect more demographic characteristics to enhance the sample’s representativeness. Meanwhile, in the future, it may be necessary to randomly select students from vocational colleges across the country as subjects for research to be more representative. Secondly, this study fails to find the exact causal relationship between variables by using a multi-time data collection design. The study in the future may use longitudinal cross-lag studies experimental designs to explore the exact causal relationship between the variables. Third, although this study has used statistical control in data processing, errors are unavoidable. The study in the future may use the experimental method and the evaluation method to control errors from the source. Fourth, the limited evidence of the validity of the measurement scales for this study, although the authors calculated values of Cronbach’s alpha and performed confirmatory analyses, there was no other evidence of validity. Meanwhile, we use only single overall scores from each survey instrument, and we do not explore the role of each dimension in detail, although this allow us to get some conclusions, it also limits the depth of this research. The study in the future may need to provide additional validity evidence for research tools, for example, conduct cognitive interviews to check that the students interpreted the survey items as intended. In addition, the study in the future can conduct a detailed analysis of the role of each dimension to draw broader conclusions. Finally, the parent-child relationship is not the only antecedent variable for individual creativity. The study in the future may explore the antecedent variables and mechanisms of creativity from multiple perspectives so as to lay the foundation for cultivating individual creativity.

## Data Availability Statement

The raw data supporting the conclusions of this article will be made available by the authors, without undue reservation.

## Ethics Statement

This study was carried out in accordance with academic ethics guidelines, and the recommendations of the Committee of History-cultural and Tourism School of Gannan Normal University, which also approved the study protocol. All subjects provided written informed consent in accordance with the Declaration of Helsinki.

## Author Contributions

YsW: conceptualization. DC: investigation and data curation. YsW and DC: methodology and writing original draft. YsW, WZ, and YbW: writing – review and editing. All authors contributed to the article and approved the submitted version.

## Conflict of Interest

The authors declare that the research was conducted in the absence of any commercial or financial relationships that could be construed as a potential conflict of interest.

## Publisher’s Note

All claims expressed in this article are solely those of the authors and do not necessarily represent those of their affiliated organizations, or those of the publisher, the editors and the reviewers. Any product that may be evaluated in this article, or claim that may be made by its manufacturer, is not guaranteed or endorsed by the publisher.
